# Factors associated with drug–drug interactions involving citalopram in the UK Biobank

**DOI:** 10.1192/bjo.2025.10060

**Published:** 2025-08-01

**Authors:** Benjamin Laplace, Win Lee Edwin Wong, Marco Menchetti, Diana De Ronchi, Paolo Fusar-Poli, Giuseppe Fanelli, MNESYS – Mood and Psychosis Sub-Project (Spoke 5), Alessandro Serretti, Cathryn M. Lewis, Chiara Fabbri

**Affiliations:** 1 Psychiatry Department of Research and Innovation, Esquirol Hospital Center, Limoges, France; 2 Social, Genetic & Developmental Psychiatry Centre, Institute of Psychiatry, Psychology & Neuroscience, King’s College London, London, UK; 3 Department of Pharmacology, Yong Loo Lin School of Medicine, National University of Singapore, Singapore, Singapore; 4 Centre for Affective Disorders, Department of Psychological Medicine, Institute of Psychiatry, Psychology & Neuroscience, King’s College London, London, UK; 5 Department of Biomedical and NeuroMotor Sciences, University of Bologna, Bologna, Italy; 6 Early Psychosis: Interventions and Clinical-detection (EPIC) Lab, Department of Psychosis Studies, Institute of Psychiatry, Psychology & Neuroscience, King’s College London, London, UK; 7 OASIS Service, South London and Maudsley NHS Foundation Trust, London, UK; 8 Department of Brain and Behavioral Sciences, University of Pavia, Pavia, Italy; 9 National Institute for Health Research Maudsley Biomedical Research Centre, South London and Maudsley NHS Foundation Trust, London, UK; 10 Department of Human Genetics, Radboud University Medical Center, Donders Institute for Brain, Cognition and Behaviour, Nijmegen, The Netherlands; 11 Department of Medicine and Surgery, Kore University of Enna, Enna, Italy; 12 Oasi Research Institute-IRCCS, Troina, Italy

**Keywords:** Antidepressants, big data, drug or substance interactions and side-effects, electronic health records, primary care

## Abstract

**Background:**

Adults with mood and/or anxiety disorders have increased risks of comorbidities, chronic treatments and polypharmacy, increasing the risk of drug–drug interactions (DDIs) with antidepressants.

**Aims:**

To use primary care records from the UK Biobank to assess DDIs with citalopram, the most widely prescribed antidepressant in UK primary care.

**Method:**

We classified drugs with pharmacokinetic or pharmacodynamic DDIs with citalopram, then identified prescription windows for these drugs that overlapped with citalopram prescriptions in UK Biobank participants with primary care records. We tested for associations of DDI status (yes/no) with sociodemographic and clinical characteristics and with cytochrome 2C19 activity, using univariate tests, then fitted multivariable models for variables that reached Bonferroni-corrected significance.

**Results:**

In UK Biobank primary care data, 25 508 participants received citalopram prescription(s), among which 11 941 (46.8%) had at least one DDI, with an average of 1.96 interacting drugs. The drugs most commonly involved were proton pump inhibitors (40% of co-prescription instances). Individuals with DDIs were more often female and older, had more severe and less treatment-responsive depression, and had higher rates of psychiatric and physical disorders. In the multivariable models, treatment resistance and markers of severity (e.g. history of suicidal and self-harm behaviours) were strongly associated with DDIs, as well as comorbidity with cardiovascular disorders. Cytochrome 2C19 activity was not associated with the occurrence of DDIs.

**Conclusions:**

The high frequency of DDIs with citalopram in fragile groups confirms the need for careful consideration before prescribing and periodic re-evaluation.

Antidepressants are recommended for the treatment of depressive and anxiety disorders; given the high prevalence of these conditions, they were used by more than 12% of the adult US population in 2013,^1^ with a slightly lower but similar rate in Europe, and evidence of an increase over time.^2,3^ For example, antidepressant prescriptions more than tripled between 1998 and 2018 in primary care in England and corresponded to 6% of all drugs dispensed in 2017.^4^ Chronic antidepressant use is common, e.g. in UK primary care, the average duration of antidepressant prescription was reported to be 4.8 years for depression and 6.8 years for anxiety and/or depression.^5^

Patients with depressive and/or anxiety disorders often take multiple medications because of frequent concomitant medical conditions, frequent chronic drug use and common psychotropic polypharmacy (affecting more than half of depressed adults).^6–8^ In patients with schizophrenia or depressive disorders being treated in hospital, adverse drug reactions (ADRs) were 2–3 times higher in those receiving polytherapy compared with those receiving monotherapy.^9^ Polypharmacy is particularly common among older adults,^10^ a group with frequent multimorbidities and chronic use of antidepressants,^11^ and the risk of drug–drug interactions (DDIs) increases with the number of prescribed drugs.^12^

DDIs are unwanted increases or decreases in drug effects caused by other medication(s) taken at the same time.^13^ DDIs have been reported to be an important cause of both hospital admissions and hospital visits, particularly when involving drugs that may be associated with gastrointestinal bleeding or cardiac rhythm alterations.^14^ Importantly, the majority of DDIs (35%) in older adults were reported to involve psychotropic medications.^15^ Previous studies have reported that DDIs are common in antidepressant users, with prevalence between 25% and 61.5%, depending on the characteristics of the sample and clinical setting.^16–18^ In patients with depression, DDIs can lead to serious ADRs such as QT-interval prolongation with cardiac arrhythmia and serotonin syndrome (a rare but potentially fatal condition); they may also reduce tolerability, treatment adherence and response.^19^ In addition to polypharmacy and age, depression itself and markers of depression severity and/or recurrence are associated with DDIs risk.^12,19,20^ Polypharmacy in older adults with depression is also associated with a low level of education and with chronic diseases, anxiety and pain.^21^

Citalopram was the most widely prescribed antidepressant in primary care in England in the period 1998–2018^4^ and is the most commonly prescribed antidepressant according to UK Biobank (UKB) primary care records.^22^ Citalopram is commonly prescribed in older adults and other fragile groups of patients,^23,24^ making it a particularly relevant drug to consider in relation to DDIs. Citalopram has been suggested as a first-line treatment for late-life depression, as it is considered to have less potential for DDIs compared with other antidepressants, but a meta-analysis suggested that there are no differences in tolerability outcomes for citalopram versus other antidepressants.^25^ Citalopram has also received a warning about its potential risk to induce QT-interval prolongation,^26^ which is one of the most frequent clinically relevant ADRs in the context of DDIs.^14^

DDIs can be divided in two main groups: pharmacokinetic and pharmacodynamic DDIs. Pharmacokinetic DDIs mainly involve interactions at the level of drug metabolism, which in the case of citalopram are substantially due to the activity of cytochrome P450 2C19 (CYP2C19).^27^ In the case of pharmacodynamic interactions, the alterations in a drug’s effect occur at the site of drug action.^13^

As citalopram is the most commonly prescribed antidepressant in primary care, and given the potential relevance of pharmacokinetic and pharmacodynamic DDIs, in this study, we aimed to compare the sociodemographic and clinical characteristics, as well as the CYP2C19 metabolic activity, of patients receiving citalopram with versus without interacting drugs, using primary care records linked to the UKB. We did not aim to find causal links in this work but to study the characteristics of participants with DDI and identify variables associated with co-prescription of citalopram and interacting drugs. These findings may suggest which categories of patients are more frequently exposed to DDIs with citalopram in primary care and the most common drugs involved in these DDIs, pointing to issues deserving clinical consideration.

## Method

### Sample

UKB is a prospective health study of ∼500 000 individuals recruited from across the UK except Northern Ireland. The main aim of the study was to identify the genetic and nongenetic determinants of diseases of middle and old age, as participants were aged between 40 and 69 years at baseline (2006–2010). UKB has collected medical history, environmental, lifestyle, multimodal imaging, genetic and other biomarker data. It combines extensive and detailed assessment of exposures with follow-up and characterisation of many different health-related outcomes. These are obtained through linkage with electronic health records such as primary care records, as well as self-reported variables.^28^

In this study, we used information included in primary care records, which were available for ∼230 000 participants. Clinical (Read v2 or CTV3) and drug codes (Read v2, BNF 2 and/or dm+d) and associated dates were available for primary care events.^29^ Citalopram prescriptions and psychiatric diagnoses extracted previously^22^ were used for this study. Further information on UKB design and data collection is available in the Supplementary Material available at https://doi.org/10.1192/bjo.2025.10060.

The UKB obtained ethics approval from the North West Multi-centre Research Ethics Committee with approval number 11/NW/0382; participants provided written informed consent before inclusion.

### Definition of DDIs involving citalopram

We considered both pharmacokinetic and pharmacodynamic DDIs. According to the Dutch Pharmacogenetics Working Group (DPWG) guidelines,^27^ citalopram is substantially metabolised by CYP2C19. Therefore, we considered pharmacokinetic DDIs to be those involving drugs that were CYP2C19 substrates, inhibitors or inducers.^30^

Pharmacodynamic DDIs were evaluated on the basis of the US Food and Drug Administration label^31^ and that of the French drug regulatory agency (Agence nationale de sécurité du médicament et des produits de santé);^32^ the latter was considered because it provides detailed information on DDIs, in particular, a classification of their clinical relevance, ranking potential DDIs on four levels: ‘Contraindicated’, ‘Not recommended’, ‘Use with caution’ and ‘To be considered’. We focused on DDIs more likely to be clinically significant; therefore, we excluded drugs belonging to the ‘To be considered’ group. Similarly, we considered DDIs with citalopram described as clinically important by the Food and Drug Administration.

A complete list of the drugs involved in DDIs with citalopram is provided in Supplementary Table 1, stratified by pharmacokinetic and pharmacodynamic interactions.

### Identification of DDIs involving citalopram in the UKB

We extracted prescription records of citalopram as in previous work.^22^ We considered as a distinct prescription window any period during which consecutive prescriptions of the drug were ≤14 weeks apart, to exclude periods when the drug was probably suspended. This was done both for citalopram and for drugs included in the DDIs list. Drugs involved in DDIs with citalopram were extracted after annotation of their chemical names with other drug names (Supplementary Table 2), to consider all possible name occurrences in the prescription records, using a case-insensitive approach.

**Fig. 1:**
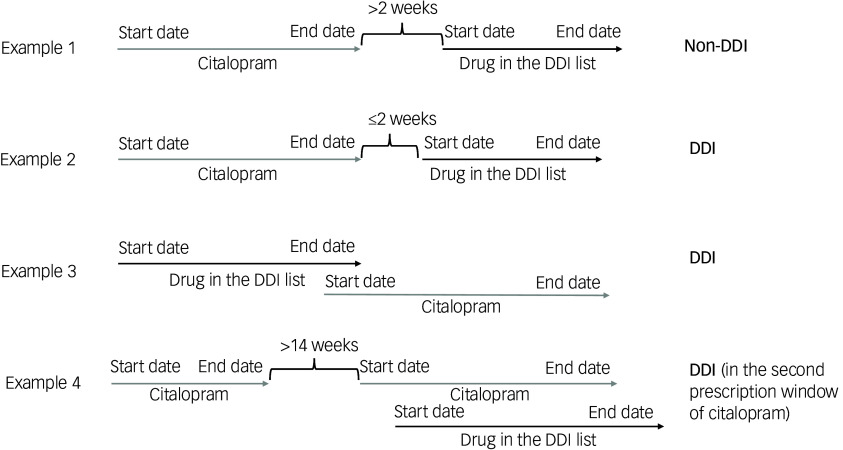
Examples of prescription windows (arrows) of citalopram (grey) and drugs in the DDI (drug–drug interaction) list. The start date is the date of the first prescription, and the end date of a prescription window is the date of the last prescription of a drug if there were no following prescriptions or the following prescription was >14 weeks apart.

We considered overlapping prescription periods between citalopram and interacting drugs when there was any time overlap between the prescription windows of the drugs ±2 weeks, to account for the treatment span after the last prescription of citalopram and the interacting drug (Fig. 1). Individuals could receive more than one drug interacting with citalopram, at the same time or in different periods; however, the outcome was binary (i.e. occurrence of at least one DDI with citalopram), as we decided to adopt a lifetime perspective. This choice was motivated by the assumption of a certain time stability in most of the variables of interest, given the age range of participants, and the difficulty of reliably estimating the timeline of multiple events using electronic health records, which reflect naturalistic clinical practice.

### Statistical analysis

We compared the characteristics of participants with at least one DDI involving citalopram and those who received citalopram but never had a co-prescription of an interacting drug (Fig. 1). The characteristics considered included age at first citalopram prescription, sex, psychiatric diagnoses, general comorbidities, treatment-resistant depression (TRD)^22^ and CYP2C19 metabolising activity.^33^ CYP2C19 metabolising activity was determined using PGxPOP, a pharmacogenetics matching engine based on PharmCAT,^34^ which uses allele definitions to characterise phenotypes (poor metabolisers, intermediate metabolisers, normal metabolisers, rapid or ultrarapid metabolisers). Individuals with undetermined or uncertain phenotypic classification were excluded. CYP2C19 was selected as the only relevant gene in relation to citalopram clinical effects, according to the Clinical Pharmacogenetics Implementation Consortium and Dutch Pharmacogenetics Working Group guidelines.^35^ A complete list of the variables and their coding is provided in Supplementary Table 3.

DDI and non-DDI groups were first compared using univariate tests (Pearson’s chi-squared test or Student’s *t*-test) as appropriate, applying a Bonferroni correction (34 variables tested, α = 0.05/34 = 1.47 × 10^−3^). Second, we used logistic regression to assess whether variables associated with DDI status in the univariate tests remained associated after adjustment for sex, age at first citalopram prescription, duration of the longest citalopram prescription, Townsend deprivation index, educational qualifications, body mass index, ethnic background and smoking history (ever smoked). The variables were selected to adjust for the possible effects of socioeconomic and demographic factors, for the length of exposure to citalopram and for possible effects of smoking on drug metabolism.^36^ The significance threshold from the univariate analyses was also applied in the regression models.

As CYP2C19 metabolic activity is more likely to affect the duration of medication co-prescription within a DDI rather than prescription of the DDI medications, for CYP2C19 activity we also tested the association with the longest co-prescription duration in each participant with a DDI, including the covariates listed above except for the longest citalopram prescription (which would be highly correlated with co-prescription duration). CYP2C19 normal metabolisers were taken as the reference group. As this was a single test based on the previous literature suggesting that reduced CYP2C19 activity (poor or intermediate metabolisers) is linked with reduced citalopram tolerability,^37^ we applied a nominal significance threshold (i.e. α = 0.05).

We performed some sensitivity analyses to test the stability of results: (a) excluding topical medications, as the probability that these have clinically relevant interactions with citalopram is lower compared with systemic routes of administration; (b) including only DDIs which are considered to be contraindicated and therefore are likely to be more clinically relevant;^32^ (c) excluding the duration of the longest citalopram prescription from the covariates, as this variable could have effects on or interactions with other independent variables (e.g. psychiatric diagnoses); and (d) replacing the covariate longest citalopram prescription with number of citalopram prescriptions, to consider an alternative measure of length of citalopram exposure.

All analyses were performed using R version 4.1.1.

## Results

### Overview of participant inclusion and prescription patterns

We included 25 508 participants who had at least one prescription of citalopram. Of these participants, 11 941 (46.8%) had at least one DDI; this reduced to 11 634 (45.6%) when we excluded DDIs involving topical medications. The median number of years covered by prescription records was 18 in both the DDI and non-DDI groups, with interquartile range (IQR) values of 13–23 and 14–23 years in the two groups, respectively. At least one diagnostic record of a depressive and/or anxiety disorder was present in 18 190 individuals (71.3%).

**Fig. 2:**
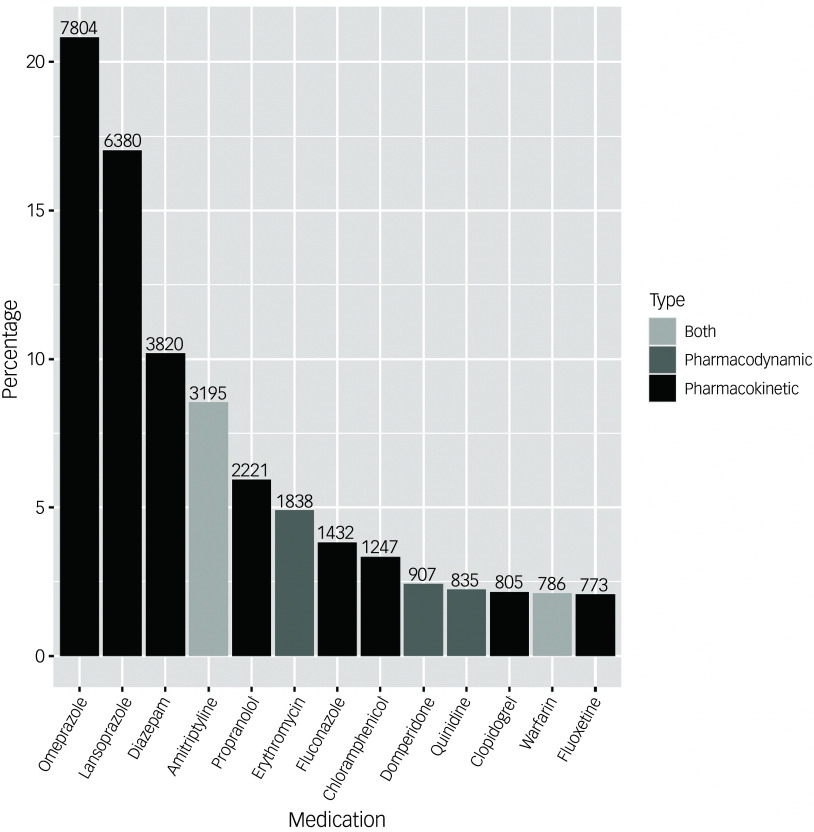
Most common medications involved in drug–drug interactions (DDIs) with citalopram. Percentage (*y*-axis) refers to the percentage for each drug calculated considering the total co-prescription instances for medications involved in DDIs (i.e. the number of times a co-prescription event occurred in the data-set), and the number reported on the top of each bar is the corresponding numerical value.

Patients in the DDI group received on average 1.96 (s.d. = 1.33; median = 1; IQR: 1–2) distinct drugs interacting with citalopram, with 3.14 (s.d. = 3.60; median = 2, IQR: 1–4) co-prescription instances, and the duration of the prescription overlap was 175 days on average, with a median of 68 days and IQR of 28–207 days (Supplementary Fig. 1). The 24% (*n* = 2897) of individuals in the DDI group had only single prescriptions (i.e. not repeated) of medications in the DDI list that overlapped with citalopram prescriptions, and these were excluded from the estimation of prescription overlap duration. The median times before the first diagnostic or prescription record and the first DDI were 17 and 10 years, respectively (IQR: 13–20 years and 5––15 years, respectively) (Supplementary Fig. 2). The most common drugs involved in DDIs with citalopram were omeprazole and lansoprazole (20.8% and 17% of all co-prescription instances, respectively), followed by diazepam (10.2%) and amitriptyline (8.5%) (Fig. 2 and Supplementary Table 4). Most co-prescriptions resulting in DDIs involved only pharmacokinetic mechanisms (74.6%), whereas 13.9% involved only pharmacodynamic mechanisms, and 11.4% involved both.

### Characteristics of the DDI group

The DDI group differed in terms of sociodemographic and clinical variables compared with the non-DDI group (Table 1). Participants in the DDI group were more often females (68.5% *v*. 65.6%), were older when receiving their first citalopram prescription (56.41 *v*. 53.59 years), received a higher number of antidepressants (3.01 *v*. 2.49) and had higher number of depression diagnostic codes (1.59 *v*. 1.50), despite showing no difference in age at first diagnosis of depression. The DDI group showed characteristics suggestive of lower socioeconomic status (e.g. lower income), had higher body mass index and increased risk of TRD (20.3% *v*. 10.7%), and were more likely to have a history of suicidal-self harm behaviours (3.1% *v*. 1.5%), as well as having increased risks of several psychiatric and non-psychiatric diseases (Table 1). We observed higher prevalences of depressive and anxiety disorders (65% *v*. 55% and 34.4% *v*. 25.6%, respectively) when looking at psychiatric diagnoses, whereas angina (4.0% *v*. 2.2%) and history of heart attack (3.9% *v*. 1.6%) in terms of general medical disorders. Other lifetime physical illnesses were more common in the DDI group, namely emphysema and/or chronic bronchitis, cancer, diabetes mellitus, high blood pressure and stroke (Table 1). There was also a higher frequency of history of a long-term illness, disability or infirmity (52.6% *v*. 37.5%) and any other serious condition (30.9% *v*. 22.0%) in the DDI group versus the non-DDI group. There was no difference in the distribution of CYP2C19 metabolising groups between participants with DDIs and those without DDIs (Table 1). The results were similar when excluding DDIs involving topical medications (Supplementary Table 5).

**Table 1:** Distribution of variables in the DDI and non-DDI groups and results of univariate tests

		DDI (*N* = 11 941)		Non-DDI (*N* = 13 567)		
**Variable^a^**	**Value**	***N* or mean (s.d.)**	**Percentage or median**	***N* or mean (s.d.)**	**Percentage or median**	***P***
**Angina** (NA = 120)	Yes	479	4.03%	290	2.15%	3.41 × 10^−18^
**Anxiety disorders** (NA = 0)	Yes	4102	34.35%	3473	25.6%	1.56 × 10^−52^
Bipolar disorders (NA = 0)	Yes	188	1.57%	158	1.16%	5.62 × 10^−3^
**Emphysema and/or chronic bronchitis** (NA = 119)	Yes	371	3.12%	271	2.01%	2.18 × 10^−8^
**Cancer** (NA = 182)	Yes	1116	9.41%	988	7.34%	2.64 × 10^−9^
CYP2C19 metabolising activity (NA = 795)	IM	2978	25.74%	3482	26.49%	3.35 × 10^−1^
	NM	4542	39.26%	5187	39.46%	
	PM	312	2.7%	361	2.75%	
	RM/UM	3737	32.3%	4114	31.3%	
**Diabetes mellitus** (NA = 150)	Yes	875	7.38%	634	4.7%	2.95 × 10^−19^
Eating disorders (NA = 0)	Yes	99	0.83%	69	0.51%	2.07 × 10^−3^
Ethnic background (NA = 127)	Other	495	4.17%	588	4.36%	4.74 × 10^−1^
	White	11387	95.83%	12911	95.64%	
**Ever smoked** (NA = 166)	Yes	7586	63.94%	8300	61.59%	1.20 × 10^−4^
**Heart attack** (NA = 120)	Yes	467	3.93%	211	1.56%	2.82 × 10^−31^
**High blood pressure** (NA = 120)	Yes	3215	27.05%	3101	22.97%	6.41 × 10^−14^
**Household income** (NA = 3994)	<18K	3600	36.32%	3223	27.78%	3.84 × 10^−52^
	>52K	1467	14.8%	2390	20.6%	
	18–31K	2584	26.07%	3005	25.9%	
	31–52K	2261	22.81%	2984	25.72%	
**Long-term illness, disability or infirmity** (NA = 919)	Yes	6054	52.63%	4902	37.46%	5.06 × 10^−126^
**Obsessive–compulsive disorder** (NA = 0)	Yes	123	1.03%	82	0.6%	1.92 × 10^−4^
**Other serious condition** (NA = 836)	Yes	3550	30.91%	2900	21.99%	6.83 × 10^−57^
Psychotic disorder	Yes	121	1.01%	99	0.73%	1.75 × 10^−2^
**Education qualifications** (NA = 362)	A levels	1220	10.37%	1562	11.67%	3.11 × 10^−48^
	College	2785	23.68%	3834	28.64%	
	None	2962	25.18%	2394	17.89%	
	O levels	2124	18.06%	2555	19.09%	
	Professional	2670	22.7%	3040	22.71%	
**Sex** (NA = 0)	Female	8180	68.5%	8902	65.62%	1.06 × 10^−6^
Sleep disorder (NA = 0)	Yes	185	1.55%	264	1.95%	1.85 × 10^−2^
Somatoform disorder (NA = 0)	Yes	548	4.59%	741	5.46%	1.66 × 10^−3^
Stress-related disorder (NA = 0)	Yes	636	5.33%	694	5.12%	4.67 × 10^−1^
**Stroke** (NA = 120)	Yes	259	2.18%	152	1.13%	4.47 × 10^−11^
**Substance and/or alcohol use disorder** (NA = 0)	Yes	493	4.13%	405	2.99%	9.09 × 10^−7^
**Suicidal and/or self-harm behaviour** (NA = 0)	Yes	368	3.08%	207	1.53%	9.42 × 10^−17^
**Treatment-resistant depression** (NA = 11184)	Yes	1461	20.27%	761	10.7%	3.29 × 10^−56^
**Depressive disorder (unipolar)** (NA = 0)	Yes	7777	65.13%	7523	55.45%	9.53 × 10^−56^
**Age at first citalopram prescription, years** (NA = 0)	Continuous	56.41 (9)	57	53.59 (8.93)	53	4.03 × 10^−137^
Age at first depression diagnosis, years (NA = 10208)	Continuous	47.90 (12.39)	49	47.77 (11.18)	48	4.85 × 10^−1^
**Body mass index** (NA = 196)	Continuous	28.41 (5.41)	27.60	27.64 (5.16)	26.87	7.01 × 10^−31^
**Number of distinct antidepressants** (NA = 0)	Continuous	3.01 (1.86)	3	2.49 (1.64)	2	8.43 × 10^−119^
**Number of distinct depression codes** (NA = 10208)	Continuous	1.59 (0.89)	1	1.50 (0.82)	1	6.27 × 10^−12^
**Longest citalopram prescription** (NA = 0)	Continuous	693.96 (900.45)	335.00	330.30 (623.71)	100	2.13 × 10^−290^
**Townsend deprivation index** (NA = 44)	Continuous	−0.70 (3.34)	−1.56	−1.11 (3.09)	−1.94	4.79 × 10^−24^

DDI, drug–drug interactions; IM, intermediate metabolisers; NM, normal metabolisers; PM, poor metabolisers; RM/UM, rapid or ultrarapid metabolisers; NA, number of missing values.

a. Number and percentage are shown for categorical variables, and mean, standard deviation and median for continuous variables. Variables with significant results after multiple testing correction are shown in bold.

When considering the association between the longest co-prescription duration and CYP2C19 activity, we found a shorter co-prescription duration in intermediate versus normal metabolisers (average 376 days, median = 116.5, IQR: 28–421 *v*. 419 days, median = 127, IQR: 28–511; *P* = 0.015). No difference was found in other metabolising groups compared with normal metabolisers, and the results were similar when excluding DDIs involving topical medications (Supplementary Table 6).

In the regression analyses (Table 2), TRD and history of suicidal-self harm behaviours had the highest effect sizes (odds ratios) for being in the DDI versus non-DDI group (odds ratio = 2.12, 95% CI: 1.91–2.34; odds ratio = 2.21, 95% CI: 1.84–2.65, respectively). Other psychiatric disorders were strongly associated with DDI status, particularly depressive disorders (odds ratio = 1.46, 95% CI: 1.38–1.54) and anxiety disorders (odds ratio = 1.53, 95% CI: 1.44–1.62) but also obsessive–compulsive disorders and substance use disorders (Table 2). The physical disorder with the highest effect size estimate with respect to DDI status was history of heart attack (odds ratio = 1.91, 95% CI: 1.60–2.28), and we also found strong associations for history of a long-term illness, disability or infirmity, and any other serious condition. Other physical disorders associated with DDI risk were history of angina, stroke and cancer. Finally, the numbers of distinct depression diagnostic codes and distinct antidepressant medications prescribed were confirmed to be associated with DDI status (Table 2). The results were similar when DDIs involving topical medications were excluded (Supplementary Table 7), as well as when we removed the duration of the longest citalopram prescription from the covariates and when we replaced it with the number of citalopram prescriptions (Supplementary Tables 8 and 9). When we restricted the analyses to the drugs with contraindicated DDIs (see ‘Statistical analysis’), 1812 participants were in the DDI group (11.78% of the sample). The most common drugs involved in these DDIs were antipsychotics, quinidine and hydroxyzine (Supplementary Fig. 3). This probably explains the higher proportion of psychotic and bipolar disorders in the DDI group versus the non-DDI group in this analysis (Supplementary Table 10A). However, the results were similar to those of the main analysis, with some additional medical comorbidities associated with DDI status in the regression analysis, in particular, emphysema and/or chronic bronchitis and diabetes (Supplementary Tables 10A, B).

**Table 2:** Multivariable regression models for outcomes in DDI (versus non-DDI) group

Variable^a^	E	s.e.	*P*	Odds ratio	Low 95% CI	High 95% CI
**Number of distinct antidepressants**	0.1876	0.008	7.49 × 10^−122^	1.2064	1.1876	1.2254
**Long-term illness, disability or infirmity**	0.4526	0.0282	9.09 × 10^−58^	1.5724	1.4878	1.6618
**Treatment-resistant depression**	0.7507	0.0517	7.78 × 10^−48^	2.1184	1.9144	2.3441
**Anxiety disorders**	0.4244	0.0294	2.61 × 10^−47^	1.5287	1.4431	1.6193
**Depressive disorders**	0.3774	0.0277	2.98 × 10^−42^	1.4585	1.3814	1.5399
**Other serious condition**	0.3566	0.0312	2.78× 10^−30^	1.4285	1.3437	1.5185
**Suicidal and/or self-harm behaviours**	0.7918	0.0936	2.63 × 10^−17^	2.2074	1.8374	2.6518
**Number of distinct depression diagnostic codes**	0.1439	0.0204	1.90 × 10^−12^	1.1548	1.1095	1.2019
**Heart attack**	0.6473	0.0902	7.24 × 10^−13^	1.9104	1.6008	2.2798
**Obsessive–compulsive disorder**	0.6136	0.1516	5.20 × 10^−5^	1.8471	1.3723	2.4862
**Substance and/or alcohol use disorders**	0.2772	0.0743	1.92 × 10^−4^	1.3194	1.1406	1.5263
**Stroke**	0.4133	0.1109	1.94 × 10^−4^	1.5118	1.2164	1.8789
**Angina**	0.2934	0.0814	3.11 × 10^−4^	1.341	1.1432	1.5729
**Cancer**	0.1603	0.0486	9.67 × 10^−4^	1.1739	1.0672	1.2912
Diabetes mellitus	0.1834	0.0593	1.99 × 10^−3^	1.2013	1.0695	1.3494
Emphysema and/or chronic bronchitis	0.1932	0.0873	2.68 × 10^−2^	1.2131	1.0223	1.4395
High blood pressure	0.0249	0.0319	4.36 × 10^−1^	1.0252	0.9631	1.0914

DDI, drug–drug interaction.

a. Variables with significant results after multiple testing correction are shown in bold.

## Discussion

In the UKB, the prevalence of DDIs involving citalopram (47%) was similar to that reported by a previous study that evaluated DDIs involving antidepressants in a population aged 65 years or older (61.5%).^17^ Notably, we found that history of TRD and self-harm and/or suicidal behaviours had strong associations with being in the DDI group, after adjustment for sociodemographic variables. The DDI group also had higher prevalences of cardiovascular disorders, cancer, and other serious and chronic conditions after adjustment for sociodemographic variables. As expected, comorbidity with severe and chronic diseases was associated with polypharmacy and therefore with risk of DDIs, as discussed in the introductory section. It remains important to underline that this is a particularly fragile group of patients, and the risk/benefit ratio of polypharmacy needs to be periodically re-evaluated.

The association between DDIs and TRD was in line with the associations of DDIs with self-harm and/or suicidal behaviours, higher number of antidepressants prescribed, and higher number of depression diagnostic records, suggesting that the difficulty in effectively treating depression and high severity in this group might have played a part in the acceptance of potential risks deriving from polytherapy. These results confirm more severe and/or recurrent disorders in those with DDIs, as found in other studies.^19,12^ However, it was not possible to determine whether the increased risk of TRD in the DDI versus the non-DDI group (20.3% *v*. 10.7%) could be explained at least in part by the higher prevalence of physical disorders, as there is good consensus that these are associated with TRD risk.^38^ Our results showed that history of cardiovascular disorders – in particular, heart attack – was strongly associated with being in the DDI group. Cardiovascular disease has been proposed to share biological mechanisms with depression, particularly through systemic inflammation, which is also involved in TRD.^39^ The evidence of lower socioeconomic status (Townsend deprivation index, qualifications and household income) in the DDI group was consistent with the previous literature.^21^ These variables are likely to act as moderators of the risk of various diseases and therefore of polypharmacy and DDIs.

According to the present study and previous work, omeprazole is among the drugs most commonly involved in DDIs with antidepressants.^20,40^ Omeprazole and other proton pump inhibitors (PPIs) such as esomeprazole and lansoprazole interact with citalopram acting as CYP2C19 inhibitors; previous studies found that citalopram serum concentrations were higher in patients treated with PPIs (e.g. +35.3% in patients co-treated with omeprazole).^41^ PPIs are very commonly prescribed medications;^42^ for example, in England, about 60 million items of PPIs were dispensed in 2018, and this number had doubled since 2008.^43^ Recent studies showed that PPIs are highly overprescribed, often with no appropriate documented indication. For example, between 25% and 70% of PPI prescriptions in the USA were reported to have no appropriate indication,^44^ and around 50% in Germany and China.^42,45^ Although it was not possible to verify whether an appropriate indication for prescribing a PPI was present in this study, we suggest that an important implication of this work is that careful clinical consideration is warranted when co-prescribing PPIs and citalopram or other antidepressants mainly metabolised by CYP2C19, particularly in fragile populations such as older adults.

We found no association between CYP2C19 metabolising activity and probability of being in the DDI versus non-DDI group. CYP2C19 activity was previously found to be associated with several proxies of citalopram efficacy or side-effects. For example, poor and intermediate metabolisers on citalopram showed increased odds of discontinuation and shorter prescription duration in UKB primary care records.^37^ Our results suggest that DDI status is not associated with CYP2C19 metabolising activity, as expected, as clinicians cannot know *a priori* CYP2C19 activity. However, physicians may adjust prescriptions based on the observed treatment tolerability, explaining our finding of shorter co-prescription periods in individuals with reduced CYP2C19 activity (intermediate metabolisers) compared with normal metabolisers.

### Limitations

These results should be interpreted considering the limitations of the study. First, UKB is not representative of the UK general population, being enriched in female, older and wealthier individuals.^46^ Second, our approach did not incorporate the temporal sequence of events (e.g. in terms of psychiatric and medical diagnoses, occurrence of TRD), and we were not able to infer causality, as this would have been beyond the scope of the study. Third, we could not determine the indication behind medication prescription, as prescription and diagnostic records are reported separately in the UKB. About 71% of included participants had a lifetime diagnosis of a depressive and/or anxiety disorder in the primary care records, suggesting that some prescriptions were motivated by another indication. Fourth, we did not consider measures of ADRs, as the naturalistic registration of these in the UKB primary care records may be associated with underreporting.^37^ Finally, this study was not intended to be comprehensive about variables associated with DDIs but to test the associations of the most common chronic physical diseases and psychiatric diagnoses and the main sociodemographic factors; the numbers of missing values were low (<5% for all variables, except for TRD, which was defined only in those with a diagnosis of depression, and household income) (Table 1).

### Clinical implications

Although severe psychopathology and treatment resistance can justify polypharmacy, this work confirms the importance of periodically checking the indication to continue a medication or to start a new medication involved in a DDI, particularly in fragile patients such as those with multiple morbidities and older adults. This information is particularly relevant for physicians most involved in multimorbidity management, such as general practitioners and geriatricians. Psychiatrists working in the consultation–liaison setting should also be aware of the potential risks of citalopram in fragile patients and perform a careful risk–benefit assessment. Key points are reporting the estimated duration of treatment and reassessing regularly the indication for continuing antidepressants, especially after the introduction of new drugs.

## Supporting information

Laplace et al. supplementary material Laplace et al. supplementary material

## Data Availability

The UKB Resource is available to bona fide researchers for health-related research in the public interest. Researchers must be registered with the UKB and be collaborators in an approved research project. Returned data and code from single publications are also available through the UKB Resource.
