# Supplementary material for: Factors associated with drug–drug interactions involving citalopram in the UK Biobank
Source: BJPsych Open. 2025 Aug 1;11(5):e166. doi: 10.1192/bjo.2025.10060 (PMC12344431; doi:10.1192/bjo.2025.10060)

Factors associated with drug-drug interactions involving citalopram in the UK Biobank

Supplementary Materials - Table of contents

Supplementary Methods 2
Supplementary Table 1 4
Supplementary Table 2 7
Supplementary Table 3 15
Supplementary Table 4 21
Supplementary Table 5 23
Supplementary Table 6 26
Supplementary Table 7 27
Supplementary Table 8 28
Supplementary Table 9 29
Supplementary Table 10 30
Supplementary Figure 1 34
Supplementary Figure 2 35
Supplementary Figure 3 36

Supplementary Methods

Characteristics of the UK Biobank cohort

UK Biobank recruited ~500K people aged between 40 and 69 years from 2006 to 2010. Assessments were undertaken in 22 centres in Scotland, England and Wales, and the baseline assessment included:

1. Written consent
2. Touch screen questionnaires
3. Face-to-face interview with a study nurse
4. Measurements, e.g., hand grip, spirometry, and bone density
5. Sample collection of blood, urine and saliva

Touch screen questionnaires included socio-demographic characteristics, lifestyle and environment, early life factors (e.g., handedness), family history, psychosocial factors (e.g., social support), health and medical history, and sex-specific factors (e.g., breast cancer screening).

In the present study, we considered several variables collected through baseline questionnaires, namely household income, qualifications, history of heart attack diagnosed by doctor, history of angina diagnosed by doctor, history of stroke diagnosed by doctor, history of high blood pressure diagnosed by doctor, history of emphysema/chronic bronchitis diagnosed by doctor, history of diabetes diagnosed by doctor, history of cancer diagnosed by doctor, long term illness, disability, or infirmity, other serious medical condition/disability diagnosed by doctor, ever smoked, ethnic background (see details for each variable in Supplementary Table 3). Townsend deprivation index was assigned to each participant at baseline based on their postcode and national census output areas. Body mass index (BMI) was part of baseline measurements. Year at birth and sex were acquired from central registry and could be updated by participants.

UK Biobank linked data to primary care records in ~230K participants (45% of the whole cohort), all of whom have provided written consent for linkage of their health-related records. The dataset contains coded clinical events (including diagnoses and procedures), prescriptions (i.e., medications prescribed but not necessarily dispensed) and a range of administrative codes (e.g. referrals to specialist hospital clinics). Non-coded, unstructured data (e.g., free-text entries) are not included (1).

As explained in a previous work (2), primary care Read v2 and CTV3 clinical codes were used to extract diagnostic information (e.g., depressive disorders, bipolar disorders, anxiety disorders, stress-related disorders, see Supplementary Table 3). Prescription records (Read v2, BNF, and/or dm+d codes) were annotated with medication chemical name, using as reference information provided by NHS websites (dm+d browser; British National Formulary). Prescription records include information on drug formulation (e.g., active ingredient concentration in case of solutions, mg of active ingredient in case of pills/tablets), while there is no field indicating daily medication dose. Each record has a corresponding date. Where clinical event or prescription date preceded or matched participant date of birth, it was in the year of their birth, or it was in the future, it has been altered to some predefined values in UKB data (01/01/1901, 02/02/1902, 03/03/1903 and 07/07/2037), and these values were set to missing in this study.

Most primary care records linked to UKB started in the '90s, and they were extracted in 2016-17 (depending on the supplier) (1), therefore they cover a timespan of about 27 years. As citalopram was marketed at the end of the '90s, almost all prescriptions (94%) in UKB primary care data occurred after 2000, and 74% of them occurred from 2006 onwards, which corresponds with UKB baseline assessment. Therefore, there was a certain alignment with UKB baseline questionnaire, and this should reduce the risk that time at assessment of baseline variables was far from the occurrence of the DDI outcome. This is in line with the age of participants at baseline and in our study (Table 1). A representation of estimated follow-up years before the first DDI with citalopram is shown in Supplementary Figure 2.

References

1. UK Biobank. Primary Care Linked Data [Internet]. Last update 2024. Available from: http://biobank.ndph.ox.ac.uk/showcase/showcase/docs/primary_care_data.pdf

2. Fabbri C, Hagenaars SP, John C, Williams AT, Shrine N, Moles L, et al. Genetic and clinical characteristics of treatment-resistant depression using primary care records in two UK cohorts. Mol Psychiatry. 2021 Jul;26(7):3363–73.

Supplementary Table 1: list of drugs with pharmacokinetic and pharmacodynamic interactions with citalopram. In terms of pharmacodynamic interactions, we considered only those classified as clinically relevant according to the French drug agency (i.e., “Contraindicated”, “Not recommended”, “Use with caution”) and those defined as “clinically important drug interactions” by the Food and Drug Administration (FDA). Both the ANSM and FDA identified as clinically relevant interactions that increase the risk of serotonin syndrome, QTc prolongation, and hemorrhagic risk. The FDA’s label also mentions the interaction with CYP2C19 inhibitors on the risk of QTc prolongation. “Contraindicated” drugs for combination with citalopram according to the French drug agency are highlighted in red.

**Pharmacokinetic interactions**			**Pharmacodynamic interactions**	
**CYP2C19 substrates**	**CYP2C19 inhibitors**	**CYP2C19 inducers**	**Drug**	**Mechanism**
Amitriptyline	Armodafinil	Efavirenz	Iproniazide	Increased risk of serotonin syndrome
Atomoxetine	Chloramphenicol	Enzalutamide	Phenelzine	Increased risk of serotonin syndrome
Brivaracetam	Cimetidine	Letermovir	Amiodarone	Increased risk of QTc prolongation
Carisoprodol	Esomeprazole	Prednisone	Amisulpride	Increased risk of QTc prolongation
Chloramphenicol	Felbamate	Rifampicin	Chloroquine	Increased risk of QTc prolongation
Clobazam	Fluoxetine	Ritonavir	Chlorpromazine	Increased risk of QTc prolongation
Clomipramine	Fluvoxamine	St. John's Wort	Crizotinib	Increased risk of QTc prolongation
Clopidogrel	Isoniazid		Cyamemazine	Increased risk of QTc prolongation
Cyclophosphamide	Ketoconazole		Disopyramide	Increased risk of QTc prolongation
Diazepam	Luliconazole		Domperidone	Increased risk of QTc prolongation
Doxepin	Modafinil		Dronedarone	Increased risk of QTc prolongation
Escitalopram	Omeprazole		Droperidol	Increased risk of QTc prolongation
Esomeprazole	Oral contraceptives		Erythromycine	Increased risk of QTc prolongation
Flibanserin	Oritavancin		Escitalopram	Increased risk of QTc prolongation and serotonin syndrome
Hexobarbital	Quercetin		Flupentixol	Increased risk of QTc prolongation
Imipramine	Ticlopidine		Fluphenazine	Increased risk of QTc prolongation
Indomethacin	Topiramate		Halofantrine	Increased risk of QTc prolongation
Labetalol	Voriconazole		Haloperidol	Increased risk of QTc prolongation
Lansoprazole	Fluconazole		Hydroquinidine	Increased risk of QTc prolongation
Moclobemide	Rucaparib		Hydroxychloroquine	Increased risk of QTc prolongation

Nelfinavir			Hydroxyzine	Increased risk of QTc prolongation
Nilutamide			Levomepromazine	Increased risk of QTc prolongation
Omeprazole			Lumefantrine	Increased risk of QTc prolongation
Ospemifene			Mequitazine	Increased risk of QTc prolongation
Pantoprazole			Methadone	Increased risk of QTc prolongation
Phenobarbitone			Moxifloxacine	Increased risk of QTc prolongation
Phenytoin			Pentamidine	Increased risk of QTc prolongation
Pomalidomide			Pimozide	Increased risk of QTc prolongation
Primidone			Pipamperone	Increased risk of QTc prolongation
Progesterone			Piperaquine	Increased risk of QTc prolongation
Proguanil			Pipotiazine	Increased risk of QTc prolongation
Propranolol			Quinidine	Increased risk of QTc prolongation
Teniposide			Sotalol	Increased risk of QTc prolongation
Tofacitinib			Spiramycine	Increased risk of QTc prolongation
Venlafaxine			Sulpiride	Increased risk of QTc prolongation
Vilazodone			Tiapride	Increased risk of QTc prolongation
Voriconazole			Toremifene	Increased risk of QTc prolongation
Warfarin			Vandetanib	Increased risk of QTc prolongation
Mavacamten			Vincamine	Increased risk of QTc prolongation
Mephobarbital			Zuclopenthixol	Increased risk of QTc prolongation
Mephenytoin			Linezolide	Increased risk of serotonin syndrome
			Moclobemide	Increased risk of serotonin syndrome
			Tedizolide	Increased risk of serotonin syndrome
			Cobimetinib	Increased hemorrhagic risk
			Amitriptyline	Increased risk of serotonin syndrome, convulsions, and other side effects
			Amoxapine	Increased risk of serotonin syndrome, convulsions, and other side effects
			Clomipramine	Increased risk of serotonin syndrome, convulsions, and other side effects

			Dosulepin	Increased risk of serotonin syndrome, convulsions, and other side effects
			Doxepine	Increased risk of serotonin syndrome, convulsions, and other side effects
			Imipramine	Increased risk of serotonin syndrome, convulsions, and other side effects
			Maprotiline	Increased risk of serotonin syndrome, convulsions, and other side effects
			Trimipramine	Increased risk of serotonin syndrome, convulsions, and other side effects
			Lithium	Increased risk of serotonin syndrome
			Tranylcypromine	Increased risk of serotonin syndrome
			Isocarboxazid	Increased risk of serotonin syndrome
			St. John's Wort	Increased risk of serotonin syndrome
			Delamanib	Increased risk of QTc prolongation
			Warfarin	Increased hemorrhagic risk

Supplementary Table 2: drugs in the DDI list (see Supplementary Table 1) annotated with other names and used for extraction from prescription records.

**Chemical name**	**Other drug names**
Amiodarone	Cordarone
Amiodarone	Nexterone
Amiodarone	Pacerone
Labetalol	Gravidol
Labetalol	Labesol
Labetalol	Labeta
Labetalol	Labetamac
Labetalol	Labil
Labetalol	Lobesol
Labetalol	Lobet
Labetalol	Normadate
Labetalol	Trandate
Labetalol	Normodyne
Amisulpride	Solian
Amisulpride	Socian
Amisulpride	Deniban
Amisulpride	Barhemsys
Lansoprazole	Prevacid
Lansoprazole	Zoton
Lansoprazole	Zapacid
Amitriptyline	Lentizol
Amitriptyline	Tryptizol
Amitriptyline	Domical
Amitriptyline	Triptafen
Levomepromazine	Nozinan
Levomepromazine	Levinan
Atomoxetine	Strattera
Atomoxetine	Atomaid
Linezolid	Zyvox
Linezolid	Linczolid
Linezolid	Lizedia
Linezolid	Pneumolid
Linezolid	Ziplemol
Linezolid	Lineurlub
Linezolid	Zyvoxid
Linezolid	Linezolida
Linezolid	Bagrizidine
Linezolid	Antizolid
Linezolid	Linezolide
Brivaracetam	Briviact

Lithium	Priadel
Lithium	Liskonum
Lithium	Camcolit
Methadone	Physeptone
Methadone	Dolophine
Methadone	Symoron
Methadone	Amidone
Methadone	Methadose
Methadone	Metadol
Methadone	Heptanon
Methadone	Heptadon
Carisoprodol	Soma
Carisoprodol	Somadril
Carisoprodol	Carisoma
Carisoprodol	Sanoma
Moclobemide	Manerix
Chloramphenicol	Optrex
Modafinil	Provigil
Chloroquine	Resochin
Chloroquine	Nivaquine
Chloroquine	Clorochina
Chloroquine	Resochina
Chloroquine	Klorokinfosfat
Chloroquine	Avloclor
Moxifloxacin	Avalox
Moxifloxacin	Avelox
Moxifloxacin	Kanavig
Moxifloxacin	Vigamox
Moxifloxacin	Xiflodrop
Moxifloxacin	Vamocin
Moxifloxacin	Lifodrox
Moxifloxacin	Flomixa
Moxifloxacin	Abimox
Moxifloxacin	Moksacin
Moxifloxacin	Ekara
Moxifloxacin	Monafox
Moxifloxacin	Floxamic
Moxifloxacin	Moxifloxacine
Moxifloxacin	Moxiflox
Chlorpromazine	Largactil
Cimetidine	Tagamet
Norethindrone	Noriday
Norethindrone	Utovlan

Norethindrone	Norethisterone
Norethindrone	Primolut
Norethindrone	Noristerat
Clobazam	Frisium
Clobazam	Tapclob
Omeprazole	Losec
Omeprazole	Omeprazol
Omeprazole	Mezzopram
Omeprazole	Omep Uno
Clomipramine	Anafranil
Pantoprazole	Pantoloc
Pantoprazole	Somac Control
Pantoprazole	Protium
Pantoprazole	Controloc
Pantoprazole	Pantecta
Pantoprazole	Pantozol
Pantoprazole	Protonix
Clopidogrel	Plavix
Clopidogrel	Iscover
Phenelzine	Nardil
Cyclophosphamide	Cytoxan
Cyclophosphamide	Cytophosphane
Cyclophosphamide	Endoxana
Cyclophosphamide	Zenew
Phenobarbital	Luminal
Phenobarbital	Phenobarbitone
Diazepam	Atensine
Diazepam	Diazemuls
Diazepam	Valium
Phenytoin	Epanutin
Disopyramide	Norpace
Disopyramide	Rythmodan
Prednisone	Meticorten
Prednisone	Delta-Cortef
Prednisone	Delta Cortef
Prednisone	Rayos
Domperidone	Motilium
Primidone	Mysoline
Dosulepin	Prothiaden
Dosulepin	Dothapax
Dosulepin	Dothiepin
Dosulepin	Dosulepine
Mavacamten	Camzyos

Mephobarbital	Methylphenobarbital
Mephobarbital	Mebaral
Mephobarbital	Mephobarbitone
Mephobarbital	Mephyltaletten
Mephobarbital	Phemiton
Mephobarbital	Prominal
Mephenytoin	Mesantoin
Fluconazole	Azocan
Fluconazole	Diflucan
Fluconazole	Canesten
Rucaparib	Rubraca
Tranylcypromine	Parnate
Isocarboxazid	Marplan
Isocarboxazid	Marplon
Isocarboxazid	Enerzer
Delamanib	Deltyba
Oral Contraceptives	Estro Progestinic
Oral Contraceptives	Estro-Progestinic
Progesterone	Crinone
Progesterone	Cyclogest
Progesterone	Lutigest
Progesterone	Utrogestan
Doxepin	Sinequan
Doxepin	Doxepine
Proguanil	Paludrine
Proguanil	Malarone
Dronedarone	Multaq
Propranolol	Inderal
Propranolol	Bedranol
Propranolol	Beta-Prograne
Propranolol	Beta Prograne
Propranolol	Half Beta-Prograne
Propranolol	Half Beta Prograne
Erythromycin	Erythrocin
Erythromycin	Erythroped
Quinidine	Quinine
Quinidine	Quinaglute
Quinidine	Quinidex
Quinidine	Cardioquin
Quinidine	Quinalan
Escitalopram	Cipralex
Rifampicin	Rifinah
Esomeprazole	Nexium

Esomeprazole	Nexium Control
Esomeprazole	Emozul
Esomeprazole	Guardium Acid Reflux
Esomeprazole	Ventra
Ritonavir	Norvir
Ritonavir	Paxlovid
Ritonavir	Kaletra
Fluoxetine	Prozac
Fluoxetine	Oxactin
Flupentixol	Depixol
Sotalol	Sotacor
Sotalol	Betapace
Fluvoxamine	Faverin
Sulpiride	Dolmatil
Sulpiride	Sulpor
Haloperidol	Dozic
Haloperidol	Haldol
Haloperidol	Serenace
Topiramate	Topamax
Hydroxychloroquine	Quinoric
Trimipramine	Surmontil
Hydroxyzine	Atarax
Hydroxyzine	Vistaril
Hydroxyzine	Ucerax
Venlafaxine	Vensir
Venlafaxine	Venaxx
Venlafaxine	Venlalic
Venlafaxine	Venlablue
Venlafaxine	Venlaneo
Venlafaxine	Venlalic
Venlafaxine	Venladex
Venlafaxine	Efexor
Venlafaxine	Vencarm
Imipramine	Tofranil
Voriconazole	Vfend
Indomethacin	Berlind
Indomethacin	Indocid
Indomethacin	Pardelprin
Warfarin	Coumadin
Isoniazid	Rifinah
Isoniazid	Rifater
Isoniazid	Rimstar

Isoniazid	Voractiv
Isoniazid	Hydra
Isoniazid	Hyzyd
Isoniazid	Isovit
Isoniazid	Laniazid
Isoniazid	Nydrazid
Isoniazid	Rimifon
Isoniazid	Stanozide
Zuclopenthixol	Clopixol
Ketoconazole	Daktarin
Ketoconazole	Dandrazol
Ketoconazole	Nizoral
Flibanserin	Addyi
Hexobarbital	Hexobarbitone
Hexobarbital	Evipan
Hexobarbital	Tobinal
Hexobarbital	Citopan
Nelfinavir	Viracept
Nilutamide	Nilandron
Nilutamide	Anandron
Ospemifene	Senshio
Ospemifene	Osphena
Pomalidomide	Pomalyst
Pomalidomide	Imnovid
Armodafinil	Nuvigil
Felbamate	Felbatol
Felbamate	Taloxa
Luliconazole	Luzu
Luliconazole	Lulicon
Luliconazole	Lulifin
Oritavancin	Tenkasi
Oritavancin	Orbactiv
Quercetin	
Ticlopidine	Ticlid
Ticlopidine	Antigreg
Ticlopidine	Aplaket
Ticlopidine	Klodin
Ticlopidine	Opteron
Ticlopidine	Ticlodone
Ticlopidine	Tiklid
Ticlopidine	Tiklyd
Efavirenz	Sustiva
Efavirenz	Stocrin

Enzalutamide	Xtandi
Letermovir	Prevymis
St John Wort	Hypericum
St John Wort	Hyperici Herba
St John Wort	Klamath Weed
St John Wort	Tipton Weed
St John Wort	Hardhay
Iproniazide	Marsilid
Iproniazide	Rivivol
Iproniazide	Iprazid
Iproniazide	Ipronid
Iproniazide	Ipronin
Crizotinib	Xalkori
Cyamemazine	Tercian
Droperidol	Inapsine
Droperidol	Droleptan
Droperidol	Dridol
Droperidol	Xomolix
Droperidol	Innovar
Fluphenazine	Motival
Halofantrine	Halfan
Hydroquinidine	Dihydroquinidine
Lumefantrine	Riamet
Lumefantrine	Coartem
Mequitazine	Primalan
Pentamidine	Pentacarinat
Pentamidine	Pentam
Pentamidine	Nebupent
Pimozide	Orap
Pipamperone	Dipiperon
Pipamperone	Dipiperal
Pipamperone	Piperonil
Pipamperone	Piperonyl
Pipamperone	Propitan
Piperaquine	Duocotecxin
Piperaquine	Artekin
Piperaquine	Eurartesim
Pipotiazine	Piportil
Spiramycine	Rovamycine
Tiapride	Tiapridal
Tiapride	Tiaprid
Tiapride	Sereprile
Tiapride	Delpral

Toremifene	Fareston
Vandetanib	Caprelsa
Vincamine	Oxybral
Vincamine	Devincan
Tedizolide	Sivextro
Tedizolide	Torezolid
Cobimetinib	Cotellic
Amoxapine	Asendis
Amoxapine	Asendin
Maprotiline	Ludiomil
Vilazodone	Viibryd
Tofacitinib	Xeljanz
Teniposide	Vumon

Supplementary Table 3: description of the variables considered in the analyses for potential association with interactions with citalopram.

**Variable**	**Field ID or source of information used**	**Original coding**	**Used coding**	**Type of variable**
Sex	31	0=female<br>1=male	0=female<br>1=male	Binary
Age at citalopram prescription	34: year of birth<br>Primary care prescription records were used to derive year at first prescription	year	Age in years: year at first citalopram prescription - year of birth	Continuous
Age at first diagnosis of depression	34: year of birth<br>Primary care diagnostic records were used to derive year of first diagnosis of depression	year	Age in years: year at first depression diagnosis - year of birth	Continuous
Duration of longer citalopram prescription	Primary care prescription records	NA	Days	Continuous
Number of distinct depression codes	Primary care prescription records	NA	Number	Continuous
Number of distinct antidepressants	Primary care prescription records, based on data extracted in previous work PMID 33753889	NA	Number	Continuous
Treatment-resistant depression	Primary care prescription records, as in previous publication, considering participants with ≥1 diagnostic record of depression PMID 33753889	NA	0=no<br>1=yes	Binary

Townsend deprivation index	22189	Number	Number	Continuous
Household income	738	1=Less than 18,000<br>2=18,000 to 30,999<br>3=31,000 to 51,999<br>4=52,000 to 100,000<br>5=Greater than 100,000<br>-1=Do not know<br>-3=Prefer not to answer	1=Less than 18,000<br>2=18,000 to 30,999<br>3=31,000 to 51,999<br>4=Greater than 52,000<br>-1=NA<br>-3=NA	Categorical (factor), 1 taken as reference
Qualifications	6138	1=College or University<br>2=A levels/AS levels or equivalent<br>3=O levels/GCSEs or equivalent<br>4=CSEs or equivalent<br>5=NVQ or HND or HNC or equivalent<br>6=Other professional qualifications<br>-7=None of the above<br>-3=Prefer not to answer	1=College/University<br>2=A/AS levels<br>3=O levels/GCSEs/CSEs<br>4=NVQ/HND/ HNC/other professional qualifications<br>5=none of the above<br>-3=NA	Categorical (factor)
Diagnosis of a depressive disorder	Primary care records, based on data extracted in previous work PMID 33753889	NA	0=no<br>1=yes	Binary
Diagnosis of an anxiety disorder	Primary care records, based on data extracted in previous work PMID 33753889	NA	0=no<br>1=yes	Binary
Diagnosis of an obsessive-compulsive disorder	Primary care records, based on data extracted in	NA	0=no<br>1=yes	Binary

	previous work PMID 33753889			
Diagnosis of a bipolar disorder	Primary care records, based on data extracted in previous work PMID 33753889	NA	0=no<br>1=yes	Binary
Diagnosis of a psychotic disorder	Primary care records, based on data extracted in previous work PMID 33753889	NA	0=no<br>1=yes	Binary
Diagnosis of drug/alcohol use disorder	Primary care records, based on data extracted in previous work PMID 33753889	NA	0=no<br>1=yes	Binary
Diagnosis of a stress-related disorder	Primary care records, based on data extracted in previous work PMID 33753889	NA	0=no<br>1=yes	Binary
Diagnosis of an eating disorder	Primary care records, based on data extracted in previous work PMID 33753889	NA	0=no<br>1=yes	Binary
Diagnosis of a somatoform disorder	Primary care records, based on data extracted in previous work PMID 33753889	NA	0=no<br>1=yes	Binary
Diagnosis of a sleep disorder	Primary care records, based on data extracted in previous work PMID 33753889	NA	0=no<br>1=yes	Binary
Self-harm suicidal behaviours	Primary care records, based on data extracted in	NA	0=no<br>1=yes	Binary

	previous work PMID 33753889			
History of heart attack diagnosed by doctor	6150	1=Heart attack<br>2=Angina<br>3=Stroke<br>4=High blood pressure<br>-7=None of the above<br>-3=Prefer not to answer	0=no heart attack (-7, 2, 3, 4)<br>1=heart attack<br>-3=NA	Binary
History of angina diagnosed by doctor	6150	1=Heart attack<br>2=Angina<br>3=Stroke<br>4=High blood pressure<br>-7=None of the above<br>-3=Prefer not to answer	0=no angina (-7, 1, 3, 4)<br>1=angina<br>-3=NA	Binary
History of stroke diagnosed by doctor	6150	1=Heart attack<br>2=Angina<br>3=Stroke<br>4=High blood pressure<br>-7=None of the above<br>-3=Prefer not to answer	0=no stroke (-7, 1, 2, 4)<br>1=stroke<br>-3=NA	Binary
History of high blood pressure diagnosed by doctor	6150	1=Heart attack<br>2=Angina<br>3=Stroke<br>4=High blood pressure<br>-7=None of the above<br>-3=Prefer not to answer	0=no high blood pressure (-7, 1, 2, 3)<br>1=high blood pressure<br>-3=NA	Binary
History of emphysema/chronic bronchitis diagnosed by doctor	6152	5=Blood clot in the leg (DVT)<br>7=Blood clot in the lung<br>6=Emphysema/chronic bronchitis<br>8=Asthma	0=no emphysema/chronic bronchitis (-7, 5, 7, 8, 9)<br>1=emphysema/chronic bronchitis<br>-3=NA	Binary

		9=Hayfever, allergic rhinitis or eczema<br>-7=None of the above<br>-3=Prefer not to answer		
History of diabetes diagnosed by doctor	2443	1=Yes<br>0=No<br>-1=Do not know<br>-3=Prefer not to answer	1=Yes<br>0=No<br>-1=NA<br>-3=NA	Binary
History of cancer diagnosed by doctor	2453	1=Yes<br>0=No<br>-1=Do not know<br>-3=Prefer not to answer	1=Yes<br>0=No<br>-1=NA<br>-3=NA	Binary
Long term illness, disability, or infirmity	100349	1=Yes<br>0=No<br>-1=Do not know<br>-3=Prefer not to answer	1=Yes<br>0=No<br>-1=NA<br>-3=NA	Binary
Other serious medical condition/disability diagnosed by doctor	2473	1=Yes<br>0=No<br>-1=Do not know<br>-3=Prefer not to answer	1=Yes<br>0=No<br>-1=NA<br>-3=NA	Binary
Ever smoked	20160	1=Yes<br>0=No	1=Yes<br>0=No	Binary
Body mass index (BMI)	21001	Number (Kg/m2)	Number (Kg/m2)	Continuous
Ethnic background	21000	1=White<br>1001=British<br>1002=Irish<br>1003=Any other white background<br>2=Mixed<br>2001=White and Black Caribbean	White (1, 1001, 1002, 1003)<br>Mixed or other	Binary

		2002=White and Black African<br>2003=White and Asian<br>2004=Any other mixed background<br>3=Asian or Asian British<br>3001=Indian<br>3002=Pakistani<br>3003=Bangladeshi<br>3004=Any other Asian background<br>4=Black or Black British<br>4001=Caribbean<br>4002=African<br>4003=Any other Black background<br>5=Chinese<br>6=Other ethnic group<br>-1=Do not know<br>-3=Prefer not to answer		
CYP2C19 metabolic activity	3388 (return ID), from previous publication PMID 33237584	Indeterminate<br>Likely Intermediate Metabolizer<br>Likely Poor Metabolizer<br>Not available<br>Poor Metabolizer<br>Intermediate Metabolizer<br>Normal metabolizer<br>Rapid Metabolizer<br>Ultrarapid Metabolizer	Indeterminate=NA<br>Likely Intermediate Metabolizer=NA<br>Likely Poor Metabolizer=NA<br>Not available=NA<br>Poor Metabolizer<br>Intermediate Metabolizer<br>Normal metabolizer<br>Rapid or Ultrarapid Metabolizer	Categorical (factor)

Supplementary Table 4: number (N) of co-prescription instances for drugs involved in DDIs with citalopram. Drugs with <10 occurrences were not reported.

**Medication**	**N**	**Percentage**
Omeprazole	7804	20.82%
Lansoprazole	6380	17.02%
Diazepam	3820	10.19%
Amitriptyline	3195	8.53%
Propranolol	2221	5.93%
Erythromycin	1838	4.90%
Fluconazole	1432	3.82%
Chloramphenicol	1247	3.33%
Domperidone	907	2.42%
Quinidine	835	2.23%
Clopidogrel	805	2.15%
Warfarin	786	2.10%
Fluoxetine	773	2.06%
Ketoconazole	719	1.92%
Esomeprazole	648	1.73%
Norethindrone	480	1.28%
Hydroxyzine	330	0.088%
Pantoprazole	320	0.85%
Dosulepin	311	0.83%
Venlafaxine	297	0.79%
Hydroxychloroquine	277	0.74%
Proguanil	241	0.64%
Escitalopram	185	0.49%
Indomethacin	151	0.40%
Cimetidine	142	0.38%
Flupentixol	140	0.37%
Progesterone	126	0.34%
Chlorpromazine	121	0.32%
Lithium	113	0.30%
Topiramate	100	0.27%
Sotalol	89	0.24%
Imipramine	67	0.18%
Phenytoin	65	0.17%
Modafinil	64	0.17%
Haloperidol	60	0.16%
Clomipramine	48	0.13%
Levomepromazine	48	0.13%
Amiodarone	43	0.11%
Primidone	42	0.11%

Amisulpride	30	0.08%
Doxepin	27	0.07%
Clobazam	24	0.06%
Trimipramine	23	0.06%
Sulpiride	16	0.04%
Rifampicin	12	0.03%
Labetalol	11	0.03%

Supplementary Table 5: results of the univariate tests when excluding topical medications from the DDI (drug-drug interaction) group. Variables in bold are significant after Bonferroni correction. NA=number of missing values. IM=intermediate metabolisers; NM=normal metabolisers; PM=poor metabolisers; RM/UM=rapid/ultrarapid metabolisers. Percentage values of variable distribution were calculated considering non-missing values, and the number with percentage of missing values are reported in a separate row for each variable.

Binary variables

		DDI (N=11,634)		no DDI (N=13,874)		
**Variable**	**Variable value**	**N**	**Percentage**	**N**	**Percentage**	**p**
**Angina**	no	11107	95.92%	13512	97.86%	3.52E-19
	yes	473	4.08%	296	2.14%	
	NA	54	0.46%	66	0.48%	
**Anxiety disorders**	no	7620	65.5%	10313	74.33%	2.66E-53
	yes	4014	34.5%	3561	25.67%	
	NA	0		0		
Bipolar disorders	no	11449	98.41%	13713	98.84%	3.72E-3
	yes	185	1.59%	161	1.16%	
	NA	0		0		
**Emphysema/chronic bronchitis**	no	11221	96.87%	13526	97.97%	3.67E-8
	yes	362	3.13%	280	2.03%	
	NA	51	0.44%	68	0.49%	
**Cancer**	no	10452	90.49%	12770	92.7%	2.18E-10
	yes	1099	9.51%	1005	7.3%	
	NA	83	0.71%	99	0.0071	
CYP2C19 metabolising activity	IM	2907	25.79%	3553	26.43%	2.72E-1
	NM	4417	39.19%	5312	39.52%	
	PM	299	2.65%	374	2.78%	
	RM/UM	3649	32.37%	4202	31.26%	
	NA	362	3.11%	433	3.12%	
**Diabetes mellitus**	no	10695	92.54%	13154	95.31%	2.01E-20
	yes	862	7.46%	647	4.69%	
	NA	77	0.66%	73	0.53%	
**Eating disorders**	no	11536	99.16%	13804	99.5%	1.18E-3
	yes	98	0.84%	70	0.05%	
	NA	0		0		
Ethnic background	other	480	4.15%	603	4.37%	4.02E-1
	white	11096	95.85%	13202	95.63	
	NA	58	0.5%	69	0.5%	
**Ever smoked**	no	4169	36.06%	5287	38.36%	1.68E-4

	yes	7392	63.94%	8494	61.64%	
	NA	73	0.63%	93	0.67%	
**Heart attack**	no	11120	96.03%	13590	98.42%	7.65E-32
	yes	460	3.97%	218	1.58%	
	NA	54	0.46%	66	0.48%	
**High blood pressure**	no	8432	72.82%	10640	77.06%	7.73E-15
	yes	3148	27.18%	3168	22.94%	
	NA	54	0.46%	66	0.48%	
**Household income**	<18K £	3516	36.44%	3307	27.87%	5.31E-53
	>52K £	1417	14.69%	2440	20.56%	
	18-31K £	2524	26.16%	3065	25.83%	
	31-52K £	2192	22.72%	3053	25.73%	
	NA	1985	17.06%	2009	14.48%	
**Long term illness, disability, or infirmity**	no	5268	47.02%	8365	62.49%	2.48E-130
	yes	5935	52.98%	5021	37.51%	
	NA	431	3.7%	488	3.52%	
**Obsessive-compulsive disorder**	no	11513	98.96%	13790	99.39%	1.44E-04
	yes	121	1.04%	84	0.61%	
	NA	0		0		
**Other serious condition**	no	7695	68.79%	10527	78.06%	5.37E-61
	yes	3491	31.21%	2959	21.94%	
	NA	448	3.85%	388	2.8%	
Psychotic disorder	no	11515	98.98%	13773	99.27%	1.36E-2
	yes	119	1.02%	101	0.73%	
	NA	0		0		
**Qualifications**	A levels	1189	10.38%	1593	11.64%	7.89E-48
	college	2705	23.61%	3914	28.59%	
	none	2895	25.27%	2461	17.98%	
	O levels	2073	18.09%	2606	19.04%	
	professional	2596	22.66%	3114	22.75%	
	NA	176	1.51%	186	1.34%	
**Sex**	female	7960	68.42%	9122	65.75%	6.65E-06
	male	3674	31.58%	4752	34.25%	
	NA	0		0		
Sleep disorder	no	11458	98.49%	13601	98.03%	6.85E-3
	yes	176	1.51%	273	1.97%	
	NA	0		0		
Somatoform disorder	no	11096	95.38%	13123	94.59%	4.58E-3

	yes	538	4.62%	751	5.41%	
	NA	0		0		
Stress-related disorder	no	11011	94.65%	13167	94.9%	3.69E-1
	yes	623	5.35%	707	5.1%	
	NA	0		0		
**Stroke**	no	11326	97.81%	13651	98.86%	4.30E-11
	yes	254	2.19%	157	1.14%	
	NA	54	0.46%	66	0.48%	
**Substance/alcohol use disorder**	no	11154	95.87%	13456	96.99%	1.84E-6
	yes	480	4.13%	418	3.01%	
	NA	0		0		
**Suicidal/self-harm behaviour**	no	11274	96.91%	13659	98.45%	1.78E-16
	yes	360	3.09%	215	1.55%	
	NA	0		0		
**Treatment-resistant depression**	no	5580	79.46%	6522	89.32%	1.86E-59
	yes	1442	20.54%	780	10.68%	
	NA	4612	39.64%	6572	47.37%	
**Depressive disorder (unipolar)**	no	4059	34.89%	6149	44.32%	7.65E-53
	yes	7575	65.11%	7725	55.68%	
	NA	0		0		

Continuous variables

Variable	DDI	Mean	SD	Median	NA	p
**Age at first citalopram prescription**	yes	56.47	8.98	57	0	4.95E-142
	no	53.60	8.93	53	0	
Age at first depression diagnosis	yes	47.88	12.41	49	4059	6.36E-1
	no	47.79	11.18	48	6149	
**BMI**	yes	28.42	5.40	27.61	107	1.85E-30
	no	27.65	5.17	26.87	89	
**N distinct antidepressants**	yes	3.03	1.87	3	0	1.44E-125
	no	2.49	1.64	2	0	
**N distinct depression codes**	yes	1.59	0.89	1	4059	4.22E-12
	no	1.50	0.82	1	6149	
**Longer citalopram prescription**	yes	692.86	902.79	332	0	1.43E-270
	no	338.29	630.56	106	0	
**Townsend deprivation index**	yes	-0.69	3.34	-1.54	16	9.78E-26
	no	-1.11	3.09	-1.95	28	

Supplementary Table 6: association between the longest co-prescription duration in participants with a DDI and CYP2C19 activity, considering all DDIs and excluding DDIs involving topical medications.

**All DDIs**				
**Comparison**	**E**	**SE**	**P**	**N of individuals in the analyses**
PM vs NM	-44.87	43.39	0.3012	3675
IM vs NM	-42.16	17.34	0.0151	5687
RM vs NM	-26.97	16.14	0.0947	6274
**DDIs excluding DDIs involving topical medications**				
**Comparison**	**E**	**SE**	**P**	**N of individuals in the analyses**
PM vs NM	-46.37	43.59	0.2874	3646
IM vs NM	-43.14	17.44	0.0134	5642
RM vs NM	-28.98	16.24	0.0744	6224

PM=poor metabolisers; IM=intermediate metabolisers; NM=normal metabolisers; RM=rapid/ultrarapid metabolisers.

Supplementary Table 7: results of the logistic regression for outcome of DDI (vs no-DDI) after excluding topical medications. Significant results after multiple-testing correction are in bold (p<1.47e-3).

Variable	E	SE	p	OR	low 95% CI	high 95% CI
**N distinct antidepressants**	0.194	0.008	2.98E-130	1.2141	1.1952	1.2333
**Long-term illness, disability, infirmity**	0.4654	0.0283	6.18E-61	1.5927	1.5067	1.6835
**Treatment-resistant depression**	0.7737	0.0514	2.92e-51	2.1678	1.9601	2.3974
**Anxiety disorder**	0.4295	0.0293	1.73E-48	1.5365	1.4507	1.6273
**Depressive disorder**	0.3701	0.0277	1.40E-40	1.4479	1.3714	1.5287
**Other serious condition**	0.3732	0.0311	4.16E-33	1.4524	1.3665	1.5437
**Suicidal/self-harm behaviour**	0.7789	0.0928	4.65E-17	2.1791	1.8167	2.6138
**Heart attack**	0.6492	0.0895	4.08E-13	1.914	1.6061	2.281
**N distinct depression diagnosis codes**	0.1465	0.0203	5.91E-13	1.1578	1.1126	1.2048
**Eating disorder**	0.7748	0.1659	3.02E-6	2.1702	1.5677	3.0041
**Obsessive-compulsive disorder**	0.6239	0.1509	3.58E-5	1.8662	1.3884	2.5084
**Angina**	0.3134	0.081	1.09E-4	1.3681	1.1672	1.6035
**Stroke**	0.4064	0.1101	2.22E-4	1.5014	1.21	1.863
**Cancer**	0.1784	0.0485	2.34E-4	1.1953	1.0869	1.3145
**Substance/alcohol use disorder**	0.2628	0.0741	3.91E-4	1.3006	1.1248	1.5039
**Diabetes mellitus**	0.1998	0.0591	7.27E-4	1.2212	1.0876	1.3711
Emphysema/chronic bronchitis	0.1856	0.0869	3.27E-2	1.2039	1.0154	1.4275
High blood pressure	0.0312	0.0319	3.28E-1	1.0317	0.9692	1.0983

Supplementary Table 8: results of the logistic regression for outcome of DDI (vs no-DDI) when excluding the duration of the longest citalopram prescription from the covariates. Significant results after multiple-testing correction are in bold (p<1.47e-3).

Variable	E	SE	p	OR	low 95% CI	high 95% CI
**N distinct antidepressants**	0.175	0.0078	3.71E-112	1.1912	1.1732	1.2096
**Long-term illness, disability, infirmity**	0.5386	0.0275	1.47E-85	1.7136	1.6237	1.8085
**Depressive disorder**	0.4511	0.027	7.22E-63	1.57	1.4891	1.6554
**Treatment-resistant depression**	0.7715	0.0503	3.54E-53	2.163	1.9601	2.3869
**Anxiety disorder**	0.4439	0.0286	2.49E-54	1.5588	1.4738	1.6486
**Other serious condition**	0.4018	0.0303	3.38E-40	1.4945	1.4083	1.586
**Suicidal/self-harm behaviour**	0.8131	0.0913	5.41E-19	2.2549	1.8854	2.6968
**Heart attack**	0.7221	0.0879	2.14E-16	2.0588	1.7329	2.4458
**N distinct depression diagnosis codes**	0.1582	0.0199	1.96E-15	1.1714	1.1266	1.218
**Substance/alcohol use disorder**	0.358	0.072	6.56E-7	1.4305	1.2422	1.6473
**Stroke**	0.5258	0.1072	9.30E-07	1.6918	1.3712	2.0874
**Angina**	0.3708	0.0788	2.56E-6	1.4489	1.2415	1.6909
**Obsessive-compulsive disorder**	0.6776	0.1471	4.11E-6	1.9691	1.4759	2.6272
**Eating disorder**	0.7177	0.1631	1.07E-5	2.0497	1.4889	2.8218
**Diabetes mellitus**	0.2504	0.0575	1.34E-5	1.2845	1.1476	1.4378
**Cancer**	0.1539	0.0473	1.15E-3	1.1664	1.0631	1.2797
Emphysema/chronic bronchitis	0.2117	0.085	1.28E-2	1.2358	1.0461	1.4598
High blood pressure	0.0496	0.031	1.09E-1	1.0509	0.9889	1.1167

Supplementary Table 9: results of the logistic regression for outcome of DDI (vs no-DDI) when replacing the covariate duration of the longest citalopram prescription with the number of citalopram prescriptions. Significant results after multiple-testing correction are in bold (p<1.47e-3).

Variable	E	SE	p	OR	low 95% CI	high 95% CI
**N distinct antidepressants**	0.1795	0.008	2.15E-111	1.1966	1.178	1.2155
**Long-term illness, disability, infirmity**	0.4448	0.0283	1.54E-55	1.5602	1.476	1.6492
**Treatment-resistant depression**	0.7265	0.0519	1.89E-44	2.0679	1.8677	2.2896
**Anxiety disorder**	0.4034	0.0295	1.33E-42	1.4969	1.4128	1.586
**Depressive disorder**	0.3459	0.0278	1.70E-35	1.4133	1.3383	1.4924
**Other serious condition**	0.3437	0.0313	5.40E-28	1.4102	1.3262	1.4994
**Suicidal/self-harm behaviour**	0.7691	0.094	2.86E-16	2.1578	1.7947	2.5944
**Heart attack**	0.645	0.0904	9.80E-13	1.906	1.5965	2.2755
**N distinct depression diagnosis codes**	0.1234	0.0206	2.04E-9	1.1313	1.0866	1.178
**Eating disorder**	0.7132	0.1678	2.13E-5	2.0405	1.4686	2.8351
**Stroke**	0.4149	0.111	1.86E-4	1.5142	1.2182	1.8822
**Obsessive-compulsive disorder**	0.5632	0.153	2.32E-4	1.7563	1.3012	2.3705
**Angina**	0.2838	0.0816	5.09E-4	1.3282	1.1319	1.5585
**Substance/alcohol use disorder**	0.2571	0.0748	5.88E-4	1.2932	1.1168	1.4974
Cancer	0.1458	0.0488	2.80E-3	1.157	1.0514	1.2731
Diabetes mellitus	0.1639	0.0596	5.99E-3	1.1781	1.0482	1.3241
Emphysema/chronic bronchitis	0.1866	0.0876	3.31E-02	1.2051	1.015	1.4309
High blood pressure	0.0265	0.032	4.08E-01	1.0269	0.9644	1.0933

Supplementary Table 10: results of the univariate analyses restricted to drugs with contraindicated DDIs with citalopram (A) and of the logistic regression for outcome of DDI (vs no-DDI) in the same group (B). Alpha value = 1.47e-3 (significant results are in bold).

A – Univariate analyses

		DDI (N=1,812)		no DDI (N=13,567)		
**variable**	**Variable value**	**N or mean (SD)**	**Percentage or median**	**N or mean (SD)**	**Percentage or median**	**p**
**Angina** (NA=69)	yes	108	5.98%	290	2.15%	1.61E-21
**Anxiety disorders** (NA=0)	yes	634	34.99%	3473	25.6%	2.73E-17
**Bipolar disorders** (NA=0)	yes	48	2.65%	158	1.16%	4.33E-7
**Emphysema/chronic bronchitis** (NA=74)	yes	91	5.04%	271	2.01%	3.16E-15
**Cancer** (NA=112)	yes	229	12.74%	988	7.34%	2.89E-15
CYP2C19 metabolising activity (NA=472)	IM	443	25.13%	3482	26.49%	5.24E-1
	NM	703	39.88%	5187	39.46%	
	PM	44	2.5%	361	2.75%	
	RM/UM	573	32.5%	4114	31.3%	
**Diabetes mellitus** (NA=87)	yes	196	10.19%	634	4.7%	1.66E-27
**Eating disorders** (NA=0)	yes	21	1.16%	69	0.51%	1.17E-3
Ethnic background (NA=76)	other	97	5.38%	588	4.36%	5.62E-2
	white	1707	94.62%	12911	95.64%	
**Ever smoked** (NA=97)	yes	1211	67.09%	8300	61.59%	6.65E-6
**Heart attack** (NA=69)	yes	111	6.14%	211	1.56%	1.04E-36
**High blood pressure** (NA=69)	yes	544	30.11%	3101	22.97%	2.68E-11
**Household income** (NA=2316)	<18K	665	45.52%	3223	27.78%	4.64E-47
	>52K	166	11.36%	2390	20.6%	

	18-31K	351	24.02%	3005	25.9%	
	31-52K	279	19.1%	2984	25.72%	
**Long term illness, disability, or infirmity** (NA=547)	yes	1160	66.48%	4902	0.3746	1.98E-118
Obsessive-compulsive disorder (NA=0)	yes	23	1.27%	82	0.006	2.09E-3
**Other serious condition** (NA=467)	yes	713	41.36%	2900	0.2199	1.72E-69
**Psychotic disorder** (NA=0)	yes	40	2.21%	99	0.0073	9.90E-10
**Education qualifications** (NA=213)	A levels	151	8.48%	1562	11.67%	5.03E-44
	College	347	19.48%	3834	28.64%	
	None	551	30.94%	2394	17.89%	
	O levels	292	16.4%	2555	19.09%	
	Professional	440	24.71%	3040	22.71%	
Sex (NA=0)	female	1255	69.26%	8902	65.62%	2.28E-3
Sleep disorder (NA=0)	yes	30	1.66%	264	1.95%	4.50E-1
Somatoform disorder (NA=0)	yes	103	5.68%	741	5.46%	7.37E-1
Stress related disorder (NA=0)	yes	105	5.79%	694	5.12%	2.43E-1
**Stroke** (NA=69)	yes	50	2.77%	152	1.13%	1.77E-8
**Substance/alcohol use disorder** (NA=0)	yes	92	5.08%	405	2.99%	3.17E-6
**Suicidal/self-harm behaviour** (NA=0)	yes	68	3.75%	207	1.53%	3.49E-11
**Treatment-resistant depression** (NA=7152)	yes	340	30.57%	761	10.7%	6.57E-73
**Depressive disorder (unipolar)** (NA=0)	yes	1232	67.99%	7523	55.45%	5.52e-24
**Age at first citalopram prescription** (NA=0)	continuous	57.46 (8.78)	58	53.59 (8.93)	53	2.40E-65

Age at first depression diagnosis (NA=10208)	continuous	47.71 (12.53)	49	47.77 (11.18)	48	0.87
**Body mass index (BMI)** (NA=196)	continuous	28.90 (5.68)	28.06	27.64 (5.16)	26.87	1.02E-18
**N distinct antidepressants** (NA=0)	continuous	3.53 (2.10)	3	2.49 (1.64)	2	2.75E-82
**N distinct depression codes** (NA=10208)	continuous	1.63 (0.95)	1	1.50 (0.82)	1	6.36E-6
**Longest citalopram prescription** (NA=0)	continuous	967.30 (1110.7)	580.5	330.30 (623.71)	100	3.70E-111
**Townsend deprivation index** (NA=44)	continuous	-0.40 (3.52)	-1.31	-1.11 (3.09)	-1.94	3.74E-16

B - logistic regression models

Variable	E	SE	p	OR	low 95% CI	high 95% CI
**N distinct antidepressants**	0.2978	0.0139	4.40E-102	1.3469	1.3107	1.3841
**Long-term illness, disability, or infirmity**	0.9236	0.059	3.56E-55	2.5183	2.2433	2.8271
**Treatment-resistant depression**	1.2265	0.0853	7.72E-47	3.4094	2.8843	4.0301
**Other serious condition**	0.7534	0.0587	1.14E-37	2.1242	1.8934	2.3832
**Depressive disorders**	0.5567	0.0582	1.18E-21	1.7449	1.5568	1.9557
**Anxiety disorders**	0.4384	0.0581	4.57E-14	1.5502	1.3834	1.7372
**Heart attack**	0.9865	0.1359	3.97E-13	2.6818	2.0547	3.5004
**Suicidal/self-harm behaviours**	1.0558	0.1578	2.22E-11	2.8743	2.1096	3.9161
**Cancer**	0.5038	0.0852	3.42E-9	1.655	1.4005	1.9558
**Diabetes mellitus**	0.5082	0.0997	3.41E-7	1.6623	1.3672	2.021
**Eating disorders**	1.3665	0.2715	4.83E-7	3.9216	2.3033	6.6768
**Bipolar disorders**	0.8792	0.1908	4.08E-6	2.409	1.6574	3.5014
**Psychotic disorders**	0.9817	0.2137	4.33E-6	2.669	1.7557	4.0574
**Angina**	0.5804	0.1308	9.17E-6	1.7868	1.3827	2.3089
**Emphysema/chronic bronchitis**	0.6011	0.1378	1.29E-5	1.8241	1.3924	2.3897
Substance/alcohol use disorders	0.3887	0.134	3.72E-3	1.4751	1.1344	1.9181
Stroke	0.5043	0.1899	7.91E-3	1.6558	1.1412	2.4025
High blood pressure	0.0675	0.0621	2.77E-1	1.0698	0.9472	1.2083

Supplementary Figure 1: duration (days) of citalopram prescription windows, DDI medications prescription windows, and co-prescription periods in individuals with at least one DDI (drug-drug interaction). Single prescriptions were excluded from the estimation of duration, as we could not determine the duration of these. In individuals with more than one citalopram prescription window or more than one DDI drug prescription window, we calculated the average duration, to avoid the inclusion of observations referred to the same individual more than once.

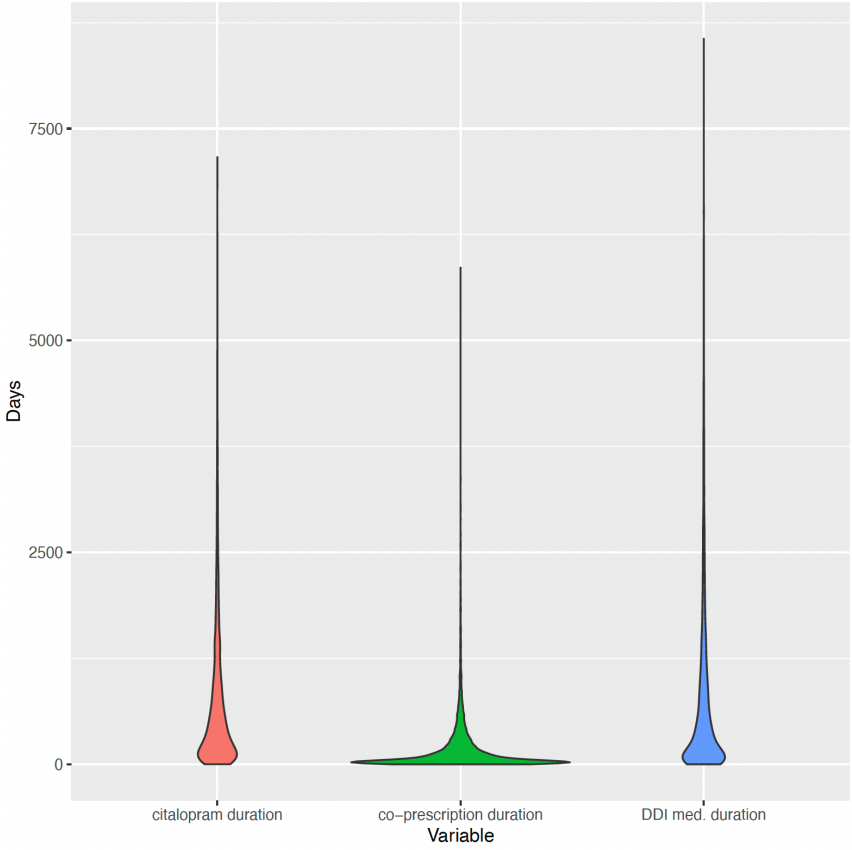

Supplementary Figure 2: number of years between the first diagnostic record (any type of diagnostic record) and the occurrence of the first DDI with citalopram (**A**), and between the first prescription record (any type of prescription) and the occurrence of the first DDI with citalopram (**B**). These figures provide an estimate of follow-up duration before the first DDI with citalopram occurred.

A

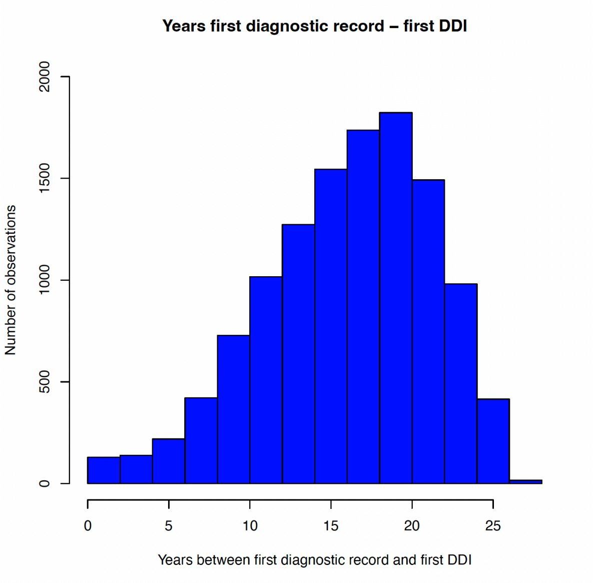

B

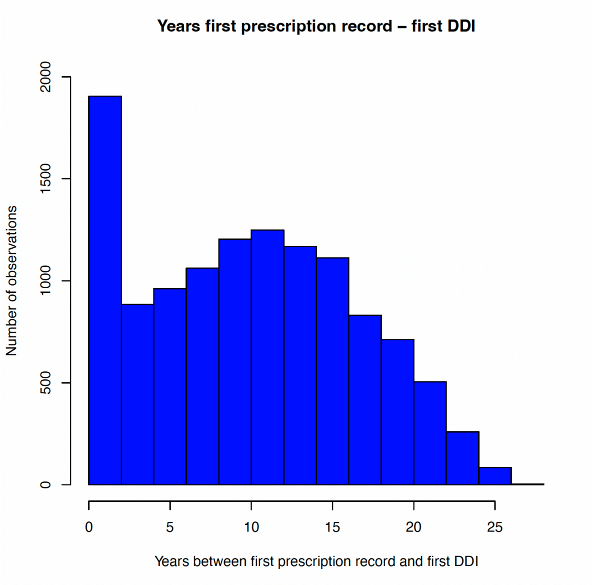

Supplementary Figure 3: most common medications involved in drug-drug interactions (DDIs) with citalopram when restricting the analysis to drugs considered as contraindicated for co-prescription with citalopram. Percentage (y axis) is referred to the percentage for each drug calculated considering the total co-prescription instances for medications involved in DDIs (i.e., number of times a co-prescription event occurred in the dataset), and the number reported on the top of each bar is referred to the corresponding numerical value.

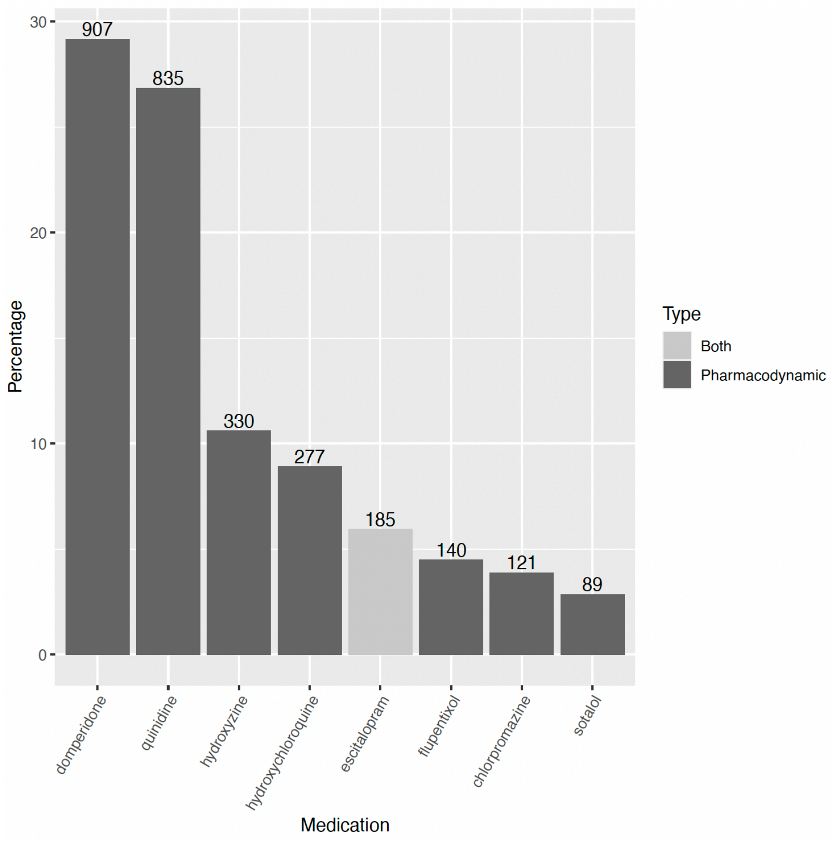

Supplement: Laplace et al. supplementary material [file S2056472425100604sup001.pdf]
